# The Teeth as Dermal Apendages

**Published:** 1887-03

**Authors:** W. D. Dunlap


### The Teeth as Dermal Apendages.

BY DR. W. D. DUNLAP.

(Read before the Alabama Dental Association.)

A strange and complicated piece of mechanism attracts our attention—a wierd and fantastic figure arises to our view. First, we see the frame work of the structure—ossific in character, and composed of many parts; hinged, double hinged, balled and socketed together—this is the frame of man. And upon this frame of bone, within and around, are to be found various structures, skillfully combined to give motion to the whole machinery, and perpetuate its powers by this means. The uncouth outline of the frame is hidden. Longitudinal, horizontal, and striated layers of tissue are piled up one on another until a symmetrical figure is produced. The whole structure is covered with a garment that is modified in structure "to suit the demands of the various parts." Within this ossific frame and outlying integuments are found a number of cavities and canals—all designed for some useful purpose, but to us the oral cavity is the most interesting; it is guarded by integuments that by muscular action close at will, and in it are the issues of life and death; through this cavity, in conjunction with the nasal openings, the air is inhaled and exhaled by the lungs. Through this cavity food passes to the stomach, both fluid and solid, and within its walls a small but complete mill is found designed to reduce the solids to a condition fit to enter the organs of digestion.

The alimentary tract is an opening extending from mouth to anus through this net work of bone and tissue—interrupted here and there by valves, coils, convolutions, sphinctors, etc., and on this line—between entrance and exit—is located the stomach. This is the great reservoir through which properly prepared supplies are disseminated through the whole structure, while the residue is voided by its own outlet.

The mill is located at the upper or highest elevation of this tract, and demands educated, yea, skilled labor to keep it in good working order. To the end that this labor be properly performed it is necessary that a knowledge should be obtained of the origin and structure of the component parts thereof, and its dependence upon the rest of the system for support and maintenance of the integrity of its various parts. With this knowledge an attempt should be made to learn how best to maintain the physical relations necessary to preserve this little mill and keep it in good order.

It will be conceded that perfect teeth in the mouth of a perfect being would need but little attention further than cleanliness. But the infirmities of humanity, both mental and physical, produce such changes in development and maintenance of integrity of structure that the little mill gets out of order and needs repair. When such is the case the services of a skilled millwright are needed. The repairs being thoroughly made, instructions should be given the miller that will enable him to use preventives with success. But in this field there is yet much to learn, and, it may be, much to unlearn. This we know, that often the best efforts to keep the teeth in good order prove failures; that hygienic rules and operative skill alike fail; that there are causes at work to bring about these failures that we do not understand, and our hope is that present and future investigations will so reveal these causes that the proper remedies may be successfully applied.

In this paper our design is to call your attention to the intimate relation that exists between the teeth and the skin, and ask that present and future attention to the subject that will enable us to determine whether pathologic conditions of the skin materially affect the teeth, and in what way.

Neligan says the skin which covers the entire external surface of the body has many uses in the animal economy; and therefore a diseased condition of it is attended, not alone with vast inconvenience to the person affected, but is also usually accompanied by a greater or less amount of derangement of the health generally. It is the organ (or medium) of touch, serves for the defense of the muscles, bones, blood vessels, and nerves placed beneath; is, moreover, the organ of absorption and excretion, and * * * agrees in many of its functions and properties with the mucous membrane, of which it is manifestly a continuation, the mucous membrane protecting the internal parts of the body, while the skin protects the external. In deranged conditions of the skin, the mucous membrane becomes more or less engaged, and in diseases which affect the mucous membrane the functions of the skin, as regards absorption and excretion, are also affected to a greater or less degree.

A. R. Robinson, M.D., speaking of hereditary syphilis, says: "Lesions of the skin usually appear; the teeth may be dwarfed or undeveloped, or easily decay as the result of syphilis; either deciduous or permanent teeth may suffer much in their nutrition and development."

Tomes says: The mucous membrane which lines the alimentary canal is continuous with—is indeed a part of the external skin, with which it blends at the lips, and that the teeth are dermal appendages."

Carpenter does not include them in his enumeration of skin appendages, but he uses the language, quoting from other authors:

"The teeth are developed from the mucous membrane covering the edges of the maxillary arches. The mucous membrane covering the edge of the jaw presents a semi-circular depression or groove; this is the primitive dental groove (of Goodsir), from the floor of which the germs of the deciduous teeth are developed, the germ of each tooth is formed by a conical elevation or papillæ of mucous membrane, which constitutes the rudimentary pulp of a milk tooth. The dental groove now becomes contracted, its margins thickened and prominent, and the groove is converted into follicles for the reception of the papillæ by the growth of septa which pass across the groove between its borders; the follicles by this means become the alveoli lined by periosteum, from the bottom of which the process of the mucous membrane of the gum rises, which is the germ of the future tooth; these changes constitute the second or follicular stage.

The papillæ now begin to grow rapidly, project from the follicles, and assume a form corresponding with that of the future teeth. The follicles soon become deeper, and from their margins small membranous processes or opercula are developed, which meeting, unite and form a lid to the new closed cavity. These processes correspond in shape to the crown of the tooth, and in number to the tubercles on its surface. The follicles of the incisor teeth have two opercula, the canines three, and the molars from four to five each. The follicles are thus converted into dental sacs, and the contained papillæ become pulps. The lips of the dental groove gradually advance over the follicles from behind forwards, and uniting gradually obliterate it. This completes the third or saccular stage."

Much of the description of the development of the teeth is here omitted, and we will proceed to quote further.

"As soon as the dental sacs are formed by the closing of the follicles they gradually enlarge, as well as their contained papillæ; each sac consists of two layers, an internal, highly vascular layer lined by epithelium, and an external areolar fibrous membrane, analagous to the corium of the mucous membrane.

The dental pulps soon become molded to the form of the future teeth, and are adherent by their bases to the bottom of the dental sacs.

Now a thin lamina or cap of dentine is formed on the most prominent point of the pulp; these lamina grow at the expense of the pulp substance, increasing in breadth and thickness.

The separate cones (if a molar tooth) ultimately coalesce, and the crown is completely formed. The pulp now becomes constricted so as to form the cervix or neck, and the remaining portion becomes elongated to form the fang.

The growth of the dentine takes place from the surface to the interior until nothing but the small pulp cavity remains in the center of the tooth—communicating by the aperture left at the point of each fang with the dental vessels and nerves.

As soon as the formation of dentine has commenced, there is developed from the inner wall of the dental sac, a soft, pulpy mass, the enamel organ, which is ultimately united to the surface of the dental pulp as its cap of dentine. It consists of a mesh of fibres, elastic and spongy, containing within its reticulaticus fluid albumen; and at the point of junction of each fibre a transparent nucleus is visible. The surface toward the dental pulp is covered by a layer of elongated nucleated cells, *the enamel membrane.* The deposition of the enamel takes place on the outer surface of the cap of dentine. The cementum appears to be formed at a later period of life by the periodental membrane extending from the margin of the enamel downward

Eruption. When the calcification of the different tissues of the tooth is sufficiently advanced to enable it to bear the pressure to which it will be afterward subjected, its eruption takes place, the tooth making its way through the gum.

The gum is absorbed by the pressure of the crown of the tooth against it, which is itself pressed up by the increasing size of the fangs. At the same time the septa between the dental sacs, at first fibrous in structure, ossify, and constitute the alveoli, there firmly embracing the necks of the teeth, and afford them a solid basis for support."

I have deemed it best to quote thus largely that we may comprehend the idea of the author, and while the evidence may not be conclusive, there are many indications that this author should have included the teeth in the list enumerated as skin appendages, as Simes and others have done.

Comparative anatomy sustains the theory, so does the loss of the teeth with the disappearance of the alveoli—through disease or old age—leaving the parts ultimately in the condition they were found when the dental groove first became perceptible, the groove even being present in many cases, and the gum as perfect as the skin is found when a scale is thrown off. If these things be so, may we not reasonably look to the skin as the immediate or incidental instrument through which the teeth are not only formed, but nourished, and that whatever tends to impair the functions of the skin may prove deleterious to the teeth also. This, too, would seem to be true from what knowledge we have of eruptive diseases and their effects upon the teeth, whether they originate in the derma or elsewhere. 'Tis true that results of defective nutrition may be witnessed in all the tissues and organs of the body, even at one time, and then the skin shares with them in this loss of nourishment; but I imagine that the skin is primarily responsible for withdrawing the supplies needed by the teeth, as evidenced in what are termed skin diseases. And in our efforts to establish this relation between skin and teeth we would not ignore the relation sustained by the blood vessels in the pulp cavity to the interior of the tooth, but would rather confine our present investigation to the source of external supplies, to the part of the structure with which we have most to do—provided the work is done in time. We might mention a number of pathological conditions of the teeth that point to the skin as the origin of the disturbance, and on the other hand, certain disturbances of the skin indicate to some extent that the teeth are defective. Who would expect to find a scrofulous subject with any but chalky and badly dissolving teeth? Should the victim escape, the offspring will surely not escape. So, too, with syphilitic taints as manifested upon the skin.

I simply desire to call your attention to this subject, deeming the relations between the skin and teeth well worth a more careful investigation than has been given the subject in the past.

